# Development and validation of a clinical risk score to predict the risk of SARS-CoV-2 infection from administrative data: A population-based cohort study from Italy

**DOI:** 10.1371/journal.pone.0237202

**Published:** 2021-01-20

**Authors:** Valentina Orlando, Federico Rea, Laura Savaré, Ilaria Guarino, Sara Mucherino, Alessandro Perrella, Ugo Trama, Enrico Coscioni, Enrica Menditto, Giovanni Corrao

**Affiliations:** 1 CIRFF, Center of Drug Utilization and Pharmacoeconomics, University of Naples Federico II, Naples, Italy; 2 Department of Pharmacy, University of Naples Federico II, Naples, Italy; 3 National Centre for Healthcare Research & Pharmacoepidemiology, at the University of Milano-Bicocca, Milan, Italy; 4 Department of Statistics and Quantitative Methods, Laboratory of Healthcare Research & Pharmacoepidemiology, Unit of Biostatistics, Epidemiology and Public Health, University of Milano-Bicocca, Milan, Italy; 5 Infectious Disease of Healthcare Direction, AORN Antonio Cardarelli, Naples, Italy; 6 Regional Pharmaceutical Unit, Campania Region, Naples, Italy; 7 Division of Cardiac Surgery, AOU San Giovanni di Dio e Ruggi d’Aragona, Salerno, Italy; University of Sassari, ITALY

## Abstract

**Background:**

The novel coronavirus (SARS-CoV-2) pandemic spread rapidly worldwide increasing exponentially in Italy. To date, there is lack of studies describing clinical characteristics of the people at high risk of infection. Hence, we aimed (i) to identify clinical predictors of SARS-CoV-2 infection risk, (ii) to develop and validate a score predicting SARS-CoV-2 infection risk, and (iii) to compare it with unspecific scores.

**Methods:**

Retrospective case-control study using administrative health-related database was carried out in Southern Italy (Campania region) among beneficiaries of Regional Health Service aged over than 30 years. For each person with SARS-CoV-2 confirmed infection (case), up to five controls were randomly matched for gender, age and municipality of residence. Odds ratios and 90% confidence intervals for associations between candidate predictors and risk of infection were estimated by means of conditional logistic regression. SARS-CoV-2 Infection Score (SIS) was developed by generating a total aggregate score obtained from assignment of a weight at each selected covariate using coefficients estimated from the model. Finally, the score was categorized by assigning increasing values from 1 to 4. Discriminant power was used to compare SIS performance with that of other comorbidity scores.

**Results:**

Subjects suffering from diabetes, anaemias, Parkinson’s disease, mental disorders, cardiovascular and inflammatory bowel and kidney diseases showed increased risk of SARS-CoV-2 infection. Similar estimates were recorded for men and women and younger and older than 65 years. Fifteen conditions significantly contributed to the SIS. As SIS value increases, risk progressively increases, being odds of SARS-CoV-2 infection among people with the highest SIS value (SIS = 4) 1.74 times higher than those unaffected by any SIS contributing conditions (SIS = 1).

**Conclusion:**

Conditions and diseases making people more vulnerable to SARS-CoV-2 infection were identified by the current study. Our results support decision-makers in identifying high-risk people and adopting of preventive measures to minimize the spread of further epidemic waves.

## Introduction

Since December 2019, the novel coronavirus (SARS-CoV-2) pandemic spread rapidly from the Hubei province in China to 185 countries causing over 3,000,000 cases [1]. The epidemic spread to and increased exponentially in Italy, earlier than in any other western Country, having generated at the current time (June 15) over 236,000 confirmed SARS-CoV-2 infections [2]. SARS-COV-2 causes a Coronavirus disease 2019 (Covid-19), for which minor symptoms are anosmia, ageusia, gastrointestinal symptoms, headache, and cutaneous manifestations and major symptoms are fever, cough, dyspnoea [3, 4]. Due to these major symptoms it may be considered necessary to hospitalize patients for respiratory complications [5].

Several hospital-based studies [6–10], including a systematic review of literature and meta-analysis [11], focused on the attempt for predicting the progression of the disease towards developing critical manifestations or death. These studies are important for the clinical practice point of view for identifying patients at whom early treatment must be guaranteed. However, as most infections are not life-threatening [7], for the public health point of view it becomes increasingly important stratifying population for identifying people at higher risk of infection. Despite this, at our best knowledge, no studies on this topic have been still published.

We therefore performed a large investigation based upon healthcare utilization database from the Italian Region of Campania aimed (1) to identify clinical predictors of the risk of SARS-CoV-2 infection, (2) to develop and validate a score overall predicting the risk of SARS-CoV-2 infection, and (3) to compare discriminant power of such a score with that from unspecific scores of clinical profile.

## Methods

### Target population and data source

Residents in Campania who were beneficiaries of the Regional Health Service (RHS) aged 30 years or older formed the target population (almost 3.9 million people, around 9% of the Italian population of that age group). Italian citizens have equal access to essential healthcare services provided by the National Health Service [12]. An automated system of healthcare utilization (HCU) databases allows managing NHS within each Italian region, including Campania. HCU data report a variety of information drawn from services provided fully or in part free of charge from NHS to beneficiaries of NHS (e.g. the ICD-CM-9 codes of inpatient diagnoses and services supplied from public or private hospitals, the ATC codes of outpatient drugs dispensed from pharmacies). This allowed to Campania Region of designing, building and routinely managing the so-called Campania Region Database (CaReDB) which formed the data source for the current study. Completeness and data validity of CaReDB being elsewhere reported [13–21]. Selected characteristics of CaReDB are described in **S1 Table**.

From the beginning of the Covid-19 epidemic, a surveillance system was implemented to detect all cases identified by reverse transcription-polymerase chain reaction (RT-PCR) testing for SARS-CoV-2. Diagnostic algorithm was based on the protocol released by the World Health Organization (WHO) [22], i.e., on nasopharyngeal swab specimens tested with at least two real-time RT PCT assays targeting different genes (E, RdRp and M) of SARS-CoV-2.

These various types of data (i.e., CaReDB and Covid-19 registry) can be interconnected, since a single individual identification code is used by all databases for each citizen enrolled. To preserve privacy, each identification code was automatically deidentified, the inverse process being allowed only to the Regional Health Authority on request from judicial authorities. Permission for using anonymized data for this study was granted to the researchers of the Centro di Ricerca in Farmacoeconomia e Farmacoutilizzazione (CIRFF) by the governance board of Unità del Farmaco della Regione Campania. According with Italian Data protection Authority, neither Ethical Committee approval, nor informed consent were required for carrying out observational studies based on HCU data as the our [23]. Our research protocol adhered to the tenets of the Declaration of Helsinki 1975 and its later amendments.

### Cases and controls

The date of SARS-CoV-2 infection diagnosis was considered as the index date and patients were extracted from the Covid-19 registry until June 10, 2020. A total of 4,629 subjects positive to SARS-CoV-2 were identified. Among these, we excluded i) patients with missing demographic information (N = 469) and ii) patients younger than 30 years at the index date (N = 663). Finally, 3,497 patients were included into the study as cases. Among them, 453 patients died during the observational period.

For each case, up to five controls were randomly selected from the target population to be matched for gender, age at index date and municipality of residence. The density incidence approach was used for selecting controls since patients who had a confirmed diagnosis of SARS-CoV-2 infection were eligible as potential controls until they became cases, and all matches had to be at risk of SARS-CoV-2 infection.

### Identifying clinical predictors of SARS-CoV-2 infection

A list of 47 diseases and conditions potentially predicting the risk of SARS-CoV-2 infection was developed starting from the lists included in several comorbidities scores, i.e., the Charlson [24], Elixhauser [25], Chronic Disease Scores [26] and RxRiskV Index [27, 28], and in some systematic reviews on Covid-19 risk factors [11, 29–31]. The algorithms for detecting patients who suffer from each of them from the above-mentioned databases were carefully chosen according to previously published papers on case-identification algorithms based on Italian healthcare data [32–36]. Individuals were classified as having one of the conditions listed if they received at least two consecutive dispensations of a drug for treatment of a specific class of disease and/or one hospital discharge with the diagnoses coded with the specific ICD-9-CM (**S2 Table**).

Conditional logistic regression was used to estimate odds ratios (ORs), with 90% confidence intervals (CIs), for the association between candidate predictors and the odds of SARS-CoV-2 infection. Predictors entered as dichotomous covariates into the model, i.e., with value 0 or 1 according to whether the specific condition was not or was recorded at least once within two-years prior baseline (2018–2019). Unadjusted and mutually adjusted models were fitted by including one by one covariate, and all covariates together, respectively. Power considerations suggested of excluding covariates with prevalence ≤ 0.12% among controls, i.e., predictors for which our sample size was not enough for detecting OR of at least 3, with a 0.80 power, and by accepting a 0.10 two-sided first type error. In addition, some conditions were grouped together when strong uncertainty of algorithm did not allow for distinguishing them.

With the aim of testing the hypothesis that predictors may affect severity of clinical manifestations of SARS-CoV-2 infection, rather than infection *per se*, analyses were restricted to strata having fatal infection. Stratifications for sex and age categories (<65 years, ≥65 years) were performed as secondary analyses.

### Developing and validating a score to predict SARS-CoV-2 infection

Seven out of ten of the 3,497 1:5 case-control sets were randomly selected to form the so-called training (derivation) set. The conditional logistic regression model was fitted to compute the ORs as above described. The least absolute shrinkage and selection operator (LASSO) method was applied for selecting the diseases / conditions able to independently predict the SARS-CoV-2 infection [37]. The coefficients estimated from the model were used for assigning a weight at each selected covariate. A weight was assigned to each coefficient by multiplying it by 10 and rounding it to the nearest whole number [38]. The weights thus obtained were then summed to generate a total aggregate score. To simplify the system, i.e., with the aim of accounting for excessive heterogeneity of the total aggregate score, the latter was categorized by assigning increasing values of 1, 2, 3 and 4 to the categories of the aggregate score of 0, 1–2, 3–4, ≥ 5, respectively. The so obtained index was denoted SARS-CoV-2 Infection Score (SIS).

Performance of SIS was explored by applying the corresponding weights to the so-called validation set consisting of the 1,048 1:5 case-control sets who did not enter the training set. To evaluate the clinical utility of SIS for predicting infection, we considered the receiver operating characteristic (ROC) curve analysis and used area under the ROC curve (AUC) as a global summary of the discriminatory capacity of the scores [39].

### Comparing specific and unspecific predictors of SARS-CoV-2 infection

Some unspecific scores surrogating general clinical profile of each case and control included into the study were considered. In particular, the number of drugs with different 3rd level ATC dispensed to, and comorbidities with different ICD-9-CM experienced by each case and control within two-years prior baseline (2018–2019) were recorded. Categorization was made by assigning increasing values of 1, 2, 3 and 4 to 0, 1–4, 5–9 and ≥10 drugs (comedication score) and 1, 2, 3 and 4 to 0, 1–2 and ≥3 comorbidities (comorbidity score). In addition, cases and controls were categorized according to the Multisource Comorbidity Score (MCS), a new index of patients’ clinical status derived from inpatients diagnostic information and outpatient drug prescriptions provided by the regional Italian data and validated for outcome prediction [40, 41]. To simplify comparisons, the original five categories of worsening clinical profile (0, 1, 2, 3 and 4) as defined by MCS, were reduced to milder (MCS = 0), middle (1≤MCS≤3) and severe (MCS≥4) categories.

With the aim of comparing discriminatory ability of specific (SIS) and unspecific (comedications, comorbidities and MCS) predictors of SARS-CoV-2 infection, ROC curves and corresponding AUCs were again used.

All analyses were performed using SAS 9.4 (Cary, NC). A 2-sided p-value of 0.10 or less was considered significant.

## Results

### Clinical predictors of SARS-CoV-2 infection

Owing to their low prevalence, fourteen conditions were excluded from this analysis (tuberculosis, weight loss, disorders involving the immune mechanisms, disorders of fluid, electrolyte and acid-base balance, coagulation defects, bipolar disorders, alcohol abuse, drug addiction, multiple sclerosis, cystic fibrosis, chronic and acute pancreatitis, anchylosing spondylitis, systemic sclerosis, systemic sclerosis). Among the 33 remaining conditions, two were grouped, i.e., chronic pulmonary obstructive disease with asthma (chronic respiratory disease), and chronic renal disease with or without dialysis.

The characteristics of the cohort members are shown in **Table 1**. Among the 31 remaining conditions, 23 (74%) showed significant association with the risk of SARS-CoV-2 infection from univariate regression.

**Table 1:** Baseline characteristics of cohort members (Covid-19 cases and related controls), individual (one by one, univariate) Odds Ratio (OR), and 90% Confidence Intervals (CI), for the relationship between selected diseases/conditions and the risk of SARS-CoV-2 infection.

	Cases (N = 3,497)	Controls (N = 17,358)	Individual OR (90% CI)
**Male gender**	1,945 (55.6%)	9,640 (55.5%)	MV
**Age (years)**			
30–64	2,375 (67.9%)	11,829 (68.1%)	MV
≥65	1,122 (32.1%)	5,538 (31.9%)	
**Infectious and parasitic diseases**			
HIV infection	68 (1.9%)	301 (1.7%)	1.12 (0.90 to 1.41)
**Neoplasms**			
Malignant neoplasms	155 (4.4%)	661 (3.8%)	1.18 (1.01 to 1.37)
**Endocrine, nutritional and metabolic diseases, and immunity disorders**			
Thyroid disorders	225 (6.4%)	920 (5.3%)	1.25 (1.10 to 1.42)
Diabetes	411 (11.8%)	1732 (10%)	1.22 (1.10 to 1.35)
Hyperlipidaemia	729 (20.8%)	3708 (21.4%)	0.97 (0.89 to 1.05)
Obesity	48 (1.4%)	153 (0.9%)	1.58 (1.20 to 2.08)
Hyperuricemia/Gout	180 (5.1%)	711 (4.1%)	1.28 (1.11 to 1.48)
**Diseases of the blood and blood-forming organs**			
Anaemias	265 (7.6%)	927 (5.3%)	1.48 (1.31 to 1.67)
**Mental disorders**			
Dementia / Alzheimer	48 (1.4%)	89 (0.5%)	2.79 (2.06 to 3.79)
Psychosis	124 (3.5%)	303 (1.7%)	2.10 (1.75 to 2.52)
Depression	233 (6.7%)	1,003 (5.8%)	1.17 (1.03 to 1.33)
Anxiety	1,369 (39.1%)	5,615 (32.3%)	1.37 (1.29 to 1.47)
**Diseases of the nervous system and sense organs**			
Parkinson’s disease	67 (1.9%)	188 (1.1%)	1.78 (1.40 to 2.26)
Epilepsy	176 (5%)	660 (3.8%)	1.35 (1.17 to 1.55)
Glaucoma	119 (3.4%)	482 (2.8%)	1.25 (1.05 to 1.48)
**Diseases of the circulatory system**			
Ischaemic Heart Disease/Angina	213 (6.1%)	841 (4.8%)	1.29 (1.12 to 1.47)
Heart failure	281 (8%)	1,005 (5.8%)	1.49 (1.31 to 1.69)
Arrhythmia	196 (5.6%)	738 (4.3%)	1.36 (1.18 to 1.57)
Valvular diseases	43 (1.2%)	180 (1%)	1.18 (0.88 to 1.57)
Vascular diseases	52 (1.5%)	186 (1.1%)	1.41 (1.08 to 1.84)
Cerebrovascular diseases	127 (3.6%)	445 (2.6%)	1.46 (1.22 to 1.74)
Hypertension	826 (23.6%)	3,731 (21.5%)	1.15 (1.07 to 1.25)
**Diseases of the respiratory system**			
Chronic respiratory diseases (COPD and asthma together)	244 (7%)	908 (5.2%)	1.37 (1.21 to 1.56)
**Diseases of the digestive system**			
Liver cirrhosis and other liver chronic diseases	54 (1.5%)	216 (1.2%)	1.23 (0.96 to 1.59)
Inflammatory bowel diseases	54 (1.5%)	169 (1%)	1.60 (1.23 to 2.07)
**Diseases of the genitourinary system**			
Kidney disease with or without dialysis	67 (1.9%)	210 (1.2%)	1.60 (1.26 to 2.03)
**Diseases of the skin and subcutaneous tissues**			
Psoriasis	23 (0.7%)	113 (0.7%)	1.02 (0.70 to 1.48)
**Diseases of the musculoskeletal system and connective tissue**			
Rheumatologic conditions	28 (0.8%)	79 (0.5%)	1.77 (1.23 to 2.56)
**Other conditions**			
Transplantation	13 (0.4%)	59 (0.3%)	1.10 (0.66 to 1.82)
Chronic pain	89 (2.5%)	378 (2.2%)	1.19 (0.97 to 1.45)
Inflammation, not elsewhere specified	410 (11.7%)	2,244 (12.9%)	0.89 (0.81 to 0.98)

Abbreviation: MV, matching variable.

**Table 2** reports multivariate association between the considered diseases/conditions and the risk of SARS-CoV-2 infection which results significant for 12 conditions (39%).

**Table 2:** Independent (all together, multivariate) Odds Ratio (OR), and 90% Confidence Intervals (CI), for the relationship between selected diseases/conditions and the risk of SARS-CoV-2 infection as a whole (3,497 cases and corresponding 17,358 controls), as well as the risk of fatal SARS-CoV-2 infection (435 cases and corresponding 2,154 controls).

	All Covid-19 cases		Fatal Covid-19 cases	
	Cases / Controls	OR (90% CI)	Cases / Controls	OR (90% CI)
**Infectious and parasitic diseases**				
HIV infection	68 / 301	1.07 (0.85 to 1.34)	11 / 47	1.04 (0.58 to 1.86)
**Neoplasms**				
Malignant neoplasms	155 / 661	0.99 (0.85 to 1.16)	35 / 147	0.99 (0.70 to 1.42)
**Endocrine, nutritional and metabolic diseases, and immunity disorders**				
Thyroid disorders	225 / 920	1.13 (0.99 to 1.29)	31 / 133	0.93 (0.64 to 1.37)
Diabetes	411 / 1,732	1.15 (1.03 to 1.28)	88 / 327	1.30 (1.01 to 1.67)
Hyperlipidaemia	729 / 3,708	0.86 (0.79 to 0.94)	131 / 703	0.69 (0.55 to 0.86)
Obesity	48 / 153	1.18 (0.89 to 1.57)	6 / 18	1.08 (0.46 to 2.56)
Hyperuricemia/Gout	180 / 711	1.08 (0.93 to 1.27)	56 / 175	1.29 (0.95 to 1.76)
**Diseases of the blood and blood-forming organs**				
Anaemias	265 / 927	1.24 (1.09 to 1.41)	63 / 184	1.45 (1.07 to 1.95)
**Mental disorders**				
Dementia / Alzheimer	48 / 89	2.14 (1.55 to 2.96)	14 / 27	1.92 (1.02 to 3.63)
Psychosis	124 / 303	1.71 (1.40 to 2.08)	35 / 57	2.68 (1.76 to 4.08)
Depression	233 / 1,003	0.98 (0.86 to 1.12)	49 / 149	1.21 (0.88 to 1.67)
Anxiety	1,369 / 5,615	1.26 (1.17 to 1.36)	217 7,824	1.33 (1.07 to 1.65)
**Diseases of the nervous system and sense organs**				
Parkinson’s disease	67 / 188	1.32 (1.02 to 1.70)	18 / 47	1.32 (0.80 to 2.18)
Epilepsy	176 / 660	1.10 (0.94 to 1.28)	44 / 99	1.57 (1.11 to 2.22)
Glaucoma	119 / 482	1.22 (1.03 to 1.46)	27 / 95	1.32 (0.89 to 1.97)
**Diseases of the circulatory system**				
Ischaemic Heart Disease/Angina	213 / 841	0.99 (0.84 to 1.15)	58 / 186	1.24 (0.88 to 1.76)
Heart failure	281 / 1,005	1.24 (1.07 to 1.44)	86 / 268	1.41 (1.04 to 1.90)
Arrhythmia	196 / 738	1.14 (0.98 to 1.33)	51 / 198	0.95 (0.68 to 1.31)
Valvular diseases	43 / 180	0.80 (0.59 to 1.09)	11 / 42	0.84 (0.44 to 1.60)
Vascular diseases	52 / 186	1.00 (0.75 to 1.32)	9 / 40	0.69 (0.36 to 1.33)
Cerebrovascular diseases	127 / 445	1.00 (0.83 to 1.21)	37 / 108	1.06 (0.72 to 1.56)
Hypertension	826 / 3,731	1.12 (1.01 to 1.24)	167 / 712	1.16 (0.94 to 1.43)
**Diseases of the respiratory system**				
Chronic respiratory diseases (COPD and asthma together)	244 / 908	1.18 (1.03 to 1.35)	50 / 178	1.14 (0.83 to 1.55)
**Diseases of the digestive system**				
Liver cirrhosis and other liver chronic diseases	54 / 216	0.93 (0.71 to 1.21)	14 / 35	1.42 (0.79 to 2.56)
Inflammatory bowel diseases	54 / 169	1.47 (1.13 to 1.91)	6 / 32	0.72 (0.33 to 1.56)
**Diseases of the genitourinary system**				
Kidney disease with or without dialysis	67 / 210	1.10 (0.84 to 1.42)	23 / 60	1.07 (0.60 to 1.90)
**Diseases of the skin and subcutaneous tissues**				
Psoriasis	23 / 113	0.93 (0.63 to 1.36)	2 / 19	0.39 (0.11 to 1.44)
**Diseases of the musculoskeletal system and connective tissue**				
Rheumatologic conditions	28 / 79	1.54 (1.06 to 2.23)	5 / 18	1.21 (0.50 to 2.91)
**Other conditions**				
Transplantation	13 / 59	0.87 (0.52 to 1.46)	3 / 8	1.45 (0.42 to 4.97)
Chronic pain	89 / 378	1.06 (0.86 to 1.31)	21 / 78	1.08 (0.69 to 1.70)
Inflammation, not elsewhere specified	410 / 2,244	0.85 (0.77 to 0.93)	74 / 316	1.12 (0.87 to 1.45)

In particular, patients suffering from diabetes, anaemias, mental disorders (dementia / Alzheimer’s disease, psychosis and anxiety), Parkinson’s disease, glaucoma, diseases of the circulatory system (heart failure and hypertension), chronic respiratory, inflammatory bowel, and rheumatologic conditions showed statistical evidence of increased risk of infection with respect to patients who did not suffer from them. Likely because of low power, only 7 conditions resulted significantly associated with the risk of fatal Covid-19 disease, but there was no relevant difference in the estimates with respect to the risk of SARS-CoV-2 infection (**Table 2**).

Anaemias, dementia/Alzheimer, psychosis, anxiety, epilepsy, heart failure, kidney diseases and particularly cystic fibrosis increased the risk of SARS-CoV-2 infection among women, whereas higher risk of infection was observed among men suffering from diabetes, psychosis, anxiety, Parkinson, arrhythmia, chronic pulmonary disease, inflammatory bowel diseases and particularly dementia/Alzheimer and rheumatologic conditions (**S3 Table**).

Estimates were similar for Covid-19 patients younger and older than 65 years. Among the former group, a significant higher risk of infection was observed for diabetes, anxiety, Parkinson’s disease, arrhythmia, inflammatory bowel and chronic pulmonary diseases, particularly dementia/Alzheimer, whereas patients older than 65 years suffering from thyroid disorders, anaemias, dementia/Alzheimer, psychosis, anxiety, epilepsy and heart failure showed a significant higher risk infection (**S4 Table**).

### SARS-CoV-2 Infection Score (SIS)

**Fig 1:**
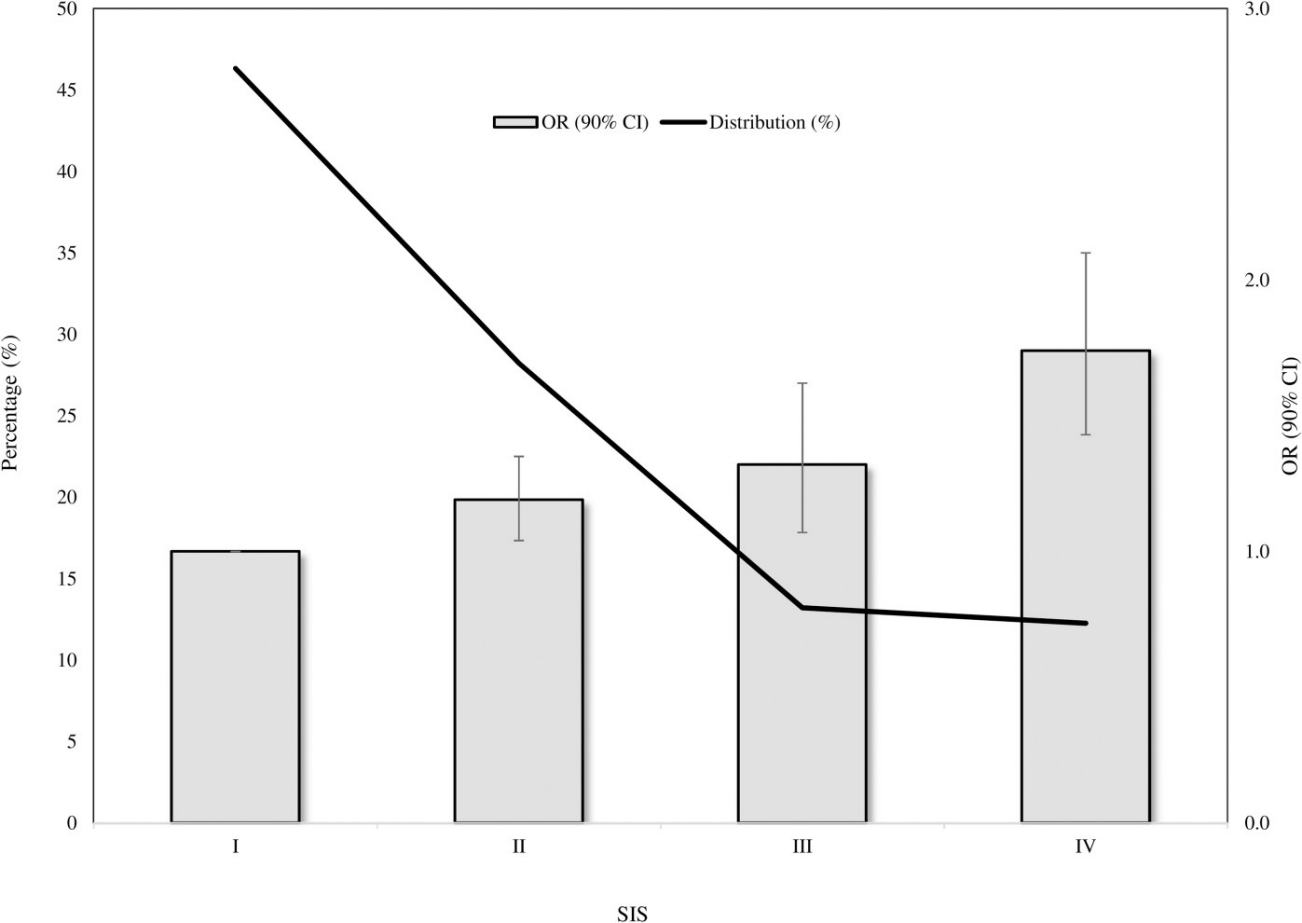
SARS-CoV-2 Infection Score (SIS) distribution among controls, and corresponding trend in odds ratios (and 90% confidence intervals) along categories of SIS. SARS-CoV-2 Infection Score: I, II, III and IV to 0, 1–2, 3–4 and ≥5.

Fifteen conditions significantly contributed to the SIS, the corresponding weights being reported in **Table 3**. Factors which most contributed to the total aggregate score were dementia / Alzheimer’s disease, kidney disease, psychosis, inflammatory bowel disease and rheumatologic conditions, while diabetes, anaemias, anxiety, Parkinson’s disease, glaucoma, heart failure, hypertension, arrhythmia, thyroid disorders and chronic respiratory disease provided small, although significant, contributions. **Fig 1** shows that, as the SIS value increases, the OR progressively increases, being the odds of SARS-CoV-2 infection among people with the highest SIS value (SIS = IV), 1.74 times higher than those unaffected by any SIS contributing conditions (SIS = I). The prevalence of controls stratified according to the SIS score gradually decreases from 50% (SIS = I) to 12% (SIS = IV).

**Table 3:** Weights, assigned to diseases that were significantly associated with the risk of Covid-19 disease, used to construct the SARS-CoV-2 Infection Score (SIS).

Disease / Condition	Log (OR)	Weights
Thyroid disorders	0.08	1
Diabetes	0.08	1
Anaemias	0.23	2
Dementia / Alzheimer	0.98	10
Psychosis	0.46	5
Anxiety	0.23	2
Parkinson’s disease	0.27	3
Glaucoma	0.15	2
Heart failure	0.27	3
Arrhythmia	0.12	1
Hypertension	0.12	1
Chronic Pulmonary disease	0.15	2
Inflammatory bowel diseases	0.40	4
Kidney dialysis	0.75	8
Rheumatologic conditions	0.55	6

### Comparing with unspecific predictors of SARS-CoV-2 infection

Generic/unspecific scores surrogating clinical profile showed to be associated with the risk of SARS-CoV-2 infection, showing patients with ≥ 10 drug treatments, those with ≥ 3 comorbidities, and those with MCS value ≥ 4, increased risk of 65%, 36% and 45% with respect to patients cotreatments, comorbidities and MCS value = I, respectively (**Table 4**).

**Table 4:** Relationship between selected score and the risk of SARS-CoV-2 infection.

Scores	OR (90% CI)
**SARS-CoV-2 Infection Score (SIS)**	
I (0)	1.00 (Ref.)
II (1–2)	1.19 (1.03 to 1.36)
III (3–4)	1.32 (1.10 to 1.58)
IV (≥5)	1.74 (1.44 to 2.10)
**Number of comedications**	
I (0)	1.00 (Ref.)
II (1–4)	1.05 (0.91 to 1.21)
III (5–9)	1.17 (0.97 to 1.41)
IV (≥10)	1.65 (1.25 to 2.19)
**Number of comorbidities**	
I (0)	1.00 (Ref.)
II (1–2)	1.21 (1.05 to 1.38)
III (≥3)	1.36 (1.15 to 1.60)
**Multisource Comorbidity Score (MCS)**	
I (0)	1.00 (Ref.)
II (1–3)	1.21 (1.03 to 1.41)
III (≥4)	1.45 (1.23 to 1.70)

**Fig 2:**
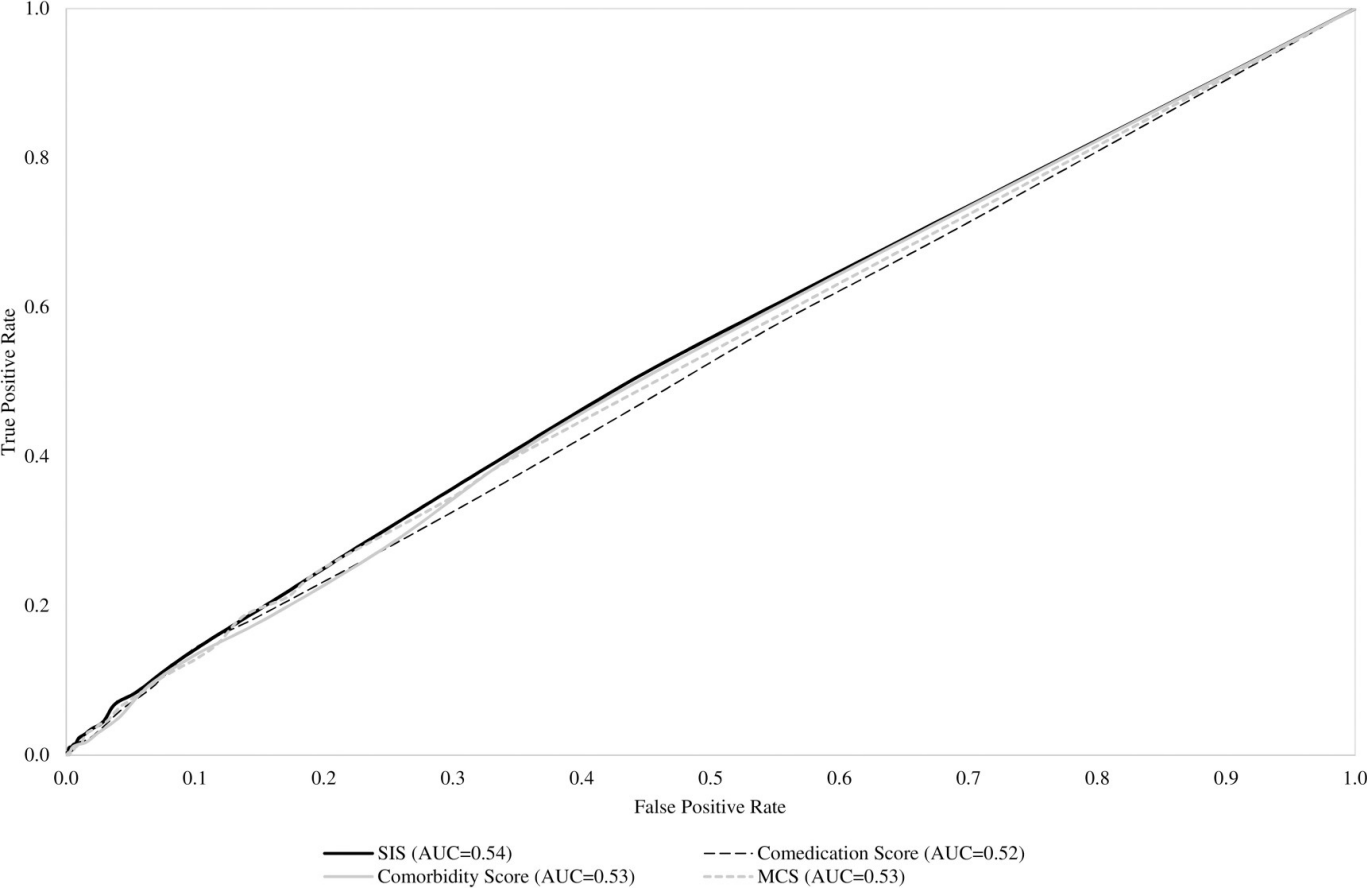
Receiver Operating Characteristics (ROC) curves comparing discriminant power of SARS-CoV-2 Infection Score (SIS), and selected unspecific score surrogating clinical profile (cotreatments, comorbidities and Multisource Comorbidity Score).

AUC (90% CI) of SIS, cotreatment and comorbidity scores and MCS respectively had values of 0.54 (0.52 to 0.56), 0.52 (0.50 to 0.54), 0.53 (0.51 to 0.55), and 0.53 (0.51 to 0.55) (**Fig 2**). There was no evidence that specific and unspecific scores had different discriminatory ability.

## Discussion

Our study shows that several diseases and conditions are significantly and independently associated with the risk of SARS-CoV-2 infection. Beyond conditions making particularly vulnerable the respiratory system (e.g., chronic obstructive pulmonary disease and asthma), comorbidities positively associated with the infection practically included all diagnostic categories. Predictors belonging to nutritional and metabolic (diabetes), cardiovascular (heart failure and hypertension) and renal diseases were widely expected, since it has accepted that SARS-CoV-2 has major implications for the cardiovascular system. Indeed, patients with heart failure [42], diabetes [43–45], hypertension [12] and kidney disease [46–48] have been consistently identified as particularly vulnerable populations, and these findings were consistently found in our study. In addition, we confirmed that people with weakened immune systems from a medical condition or treatment are at a higher risk. Among these, those living with haemoglobin disorders [49], inflammatory bowel disease [50] and immune-rheumatological diseases [51] must be considered vulnerable groups for SARS-CoV-2 infection. Mental health and cognitive function might have independent utility in understanding the burden of respiratory disease, since they may influence the risk of contracting the infection, at least in part by impairing innate or adaptive immunity [52] and diminishing the precautions taken to minimize risk. Another explanation of our findings is that people with history of depression [53], psychosis [54] and stress disorders [55] could experience elevated rates of an array of respiratory infections because these conditions often require treatment in a psychiatric care facility, and the risk of infection can be particularly high in these structures. Finally, our study adds evidence regarding the impact of diseases and conditions on the risk of SARS-CoV-2 infection between men and women. As pointed out by a recent study [56], sex and age disaggregated data are essential for understanding the distributions of risk infection in the population and the extent to which they affect clinical outcomes.

Despite our results confirm that a wide range of diseases and conditions likely increase vulnerability to SARS-CoV-2 infection, and probably its more severe clinical manifestations, we have not been able to develop a score that accurately may predict the risk of infection. In addition, we found that predictive ability of the score obtained by weighting risk factors of SARS-CoV-2 infection did not overcome that of some generic scores of comorbidities and comedications. This expands upon previous findings of individual comorbidities as independent risk factors for SARS-CoV-2 infection [57, 58], and confirms our substantial inability to predict the risk of SARS-CoV-2 infection. This can be explained by several limitations of our approach, which generate estimates biased towards the null. First, exposure misclassification regards our inability to careful capturing conditions and diseases through algorithms based on healthcare utilization databases [59]. Second, it is well known that outcome misclassification can bias epidemiologic results. For Covid-19, suboptimal test sensitivity, despite excellent specificity, results in an overestimation of cases in the early stages of an outbreak, and substantial underestimation of cases as prevalence increases [60]. It should be noticed, however, that both, exposure and outcome misclassification likely drew estimates towards the null (i.e., underestimate the strength of the association between their presence and the outcome risk) so generating uncertainty for the weighting approach of score developing. Third, the lack of information on biologic markers potentially able to predict infection, and severity of its clinical manifestations, is another limitation of our study. For example, according to the current literature, some laboratory hallmarks have been shown to predict infection, particularly in more severe cases [61]. Finally, our choice of accepting a 0.10 first type error, and of consequently reporting 90% confidence intervals, is justified by the exploratory nature of our study, but at the same time likely generate false positive signals, so limiting discriminant power of the score.

Three other elements of weakness should be acknowledged. First, the lack of data regarding the clinical outcome experienced by SARS-CoV-2 positive patients in terms of home isolation, hospitalization and admission in intensive care. Second, because few people aged less than 30 years were diagnosed to be affected by SARS-CoV-2 infection during the investigated period, and few of them suffered from chronic conditions such as those considered in our study, patients with less than 30 years were excluded from the analysis. Although this reduced the uncertainty of the results, the generalisability of our findings requires extreme caution. Finally, because data on stays in long-term facilities are not recorded in our database, we cannot exclude that the higher risks associated with mental disorders observed in our study could be explained by confounding, i.e., patients who suffered from these conditions are often hospitalized in these structures where the risk of infection can be particularly high.

In conclusion, taking the limitations we discussed into account, we identified conditions and diseases that make people more vulnerable to SARS-CoV-2 infection. These findings contribute to inform public health, and clinical decisions regarding risk stratifying. However, further research is need for developing a score reliably predicting the risk, possibly by integrating healthcare utilization with clinical and biological data.

Our results can be an important tool supporting all clinical and political stakeholders allowing the identification of the population most at risk of contracting SARS-CoV-2 infection and facilitating the provision of appropriate preventive/therapeutic measures, especially with the hypothetic prediction of a new autumn outbreak. Adopting preventive measures can help to minimize the damage generated by a potential new relapse that the health systems will face.

## Supporting information

S1 Table Campania Region Database (CaReDB) characteristics. ATC = Anatomical Therapeutic Chemical; ICD-9-CM = *International Classification of Diseases*, 9th Revision, Clinical Modification. ^a^Time span covered was 2009–2018 for hospital-discharge records and 2014–2019 for outpatient pharmacy records.
(DOCX)

S2 Table List of diseases and conditions candidate for predicting SARS-CoV-2 infection, and corresponding ICD-CM and ATC codes used for detecting they. (DOCX)

S3 Table Odds Ratio (OR), and 90% Confidence Intervals (CI), for the relationship between selected diseases/conditions and the risk of SARS-CoV-2 infection, stratified according to gender. (DOCX)

S4 Table Odds Ratio (OR), and 90% Confidence Intervals (CI), for the relationship between selected diseases/conditions and the risk of SARS-CoV-2 infection, stratified according to age categories (i.e., younger and older 65 years). (DOCX)
