# Supplementary material for: Development and validation of a clinical risk score to predict the risk of SARS-CoV-2 infection from administrative data: A population-based cohort study from Italy
Source: PLoS One. 2021 Jan 20;16(1):e0237202. doi: 10.1371/journal.pone.0237202 (PMC7816996; doi:10.1371/journal.pone.0237202)
Supplement: S1 Table — ATC = Anatomical Therapeutic Chemical; ICD-9-CM = International Classification of Diseases, 9th Revision, Clinical Modification. aTime span covered was 2009–2018 for hospital-discharge records and 2014–2019 for outpatient pharmacy records. (DOCX) [file pone.0237202.s001.docx]

**S1 Table.** **Campania Region Database (CaReDB) characteristics.**

| Characteristics |  |
| --- | --- |
| Geographic area | Campania, Italy |
| Population covered | ~5.8 million inhabitants |
| Age span covered | Whole population |
| Time span covered ^a^ | 2010–2019 |
| Scope | Drug utilisation and outcome research; real-world evidences for public health; pharmacoepidemiologic and pharmacoeconomic analysis |
| Data sources | Specific fields in the data sources contributing to a database |
| Demographic information | Patient ID; sex; date of birth; municipality of residence; district; local health unit |
| Outpatient pharmacy records | Patient ID; drug code; prescription date; delivery date; quantity; ATC code; price; defined daily dose; drug distribution channel (file F, file D) |
| Hospital-discharge records | Patient ID; type of admission; date of admission; reasons for discharge; diagnoses (ICD-9 code); procedures (ICD-9 code); date of discharge; disease-related group |
| Type of codes for diagnoses | Hospital: ICD-9-CM |
| Type of codes for medications | ATC classification |

ATC = Anatomical Therapeutic Chemical; ICD-9-CM = *International Classification of Diseases*, 9th Revision, Clinical Modification.

^a^ Time span covered was 2009–2018 for hospital-discharge records and 2014–2019 for outpatient pharmacy records.
