# Supplementary material for: Development and validation of a clinical risk score to predict the risk of SARS-CoV-2 infection from administrative data: A population-based cohort study from Italy
Source: PLoS One. 2021 Jan 20;16(1):e0237202. doi: 10.1371/journal.pone.0237202 (PMC7816996; doi:10.1371/journal.pone.0237202)
Supplement: S2 Table — (DOCX) [file pone.0237202.s002.docx]

**S2 Table. List of diseases and conditions candidate for predicting SARS-CoV-2 infection, and corresponding ICD-CM and ATC codes used for detecting they.**

|  |  | **ICD-9 CM code** | **ATC code** |
| --- | --- | --- | --- |
| ID |  |  |  |
|  | **Infectious and parasitic diseases** |  |  |
| 1 | HIV infection | 042.x-044.x | J01FA09, J05AF01, J05AR01, J05AR04, J05AR05, J05AF02, J05AF03, J05AE01, J05AE08, J05AF11, J05AG01, J05AG02, J05AG03, J05AR06, J05AX07, P01CX01, P01AX06 |
| 2 | Tuberculosis | 010.x - 018.x | J04AB |
|  | **Neoplasms** |  |  |
| 3 | Malignant Neoplasms | 140.x-165.x, 170.x-176.x, 179.x-199.x, 200.x-208-x | L01, L03AC, L02BA01, L02BA02, L02BG02, L02BG03, L02BG04, L02BG06, L02BB01, L02BB03, L02AE02, L02AE04, L02AB01 |
|  | **Endocrine, nutritional and metabolic diseases, and immunity disorders** |  |  |
| 4 | Thyroid disorders | 240.x-246.x, 252.1, 252.0 | H03A, H03B |
| 5 | Diabetes | 250.x | A10 |
| 6 | Hyperlipidaemia | 272.2, 272.4 | C10 |
| 7 | Obesity | 278.0x | - |
| 8 | Weight loss | 260-263.x | - |
| 9 | Disorders of fluid, electrolyte, and acid-base balance | 276.x | - |
| 10 | Hyperuricemia/Gout | 274.x | M04AC01, M04AA, M04AB |
| 11 | Disorders involving the immune mechanisms | 279.x | - |
|  | **Diseases of the blood and blood-forming organs** |  |  |
| 12 | Coagulation defects | 286.x | B02B |
| 13 | Anaemias | 280.x-285.x | B03A, B03B, B03XA01, L03AA |
|  | **Mental disorders** |  |  |
| 14 | Dementia / Alzheimer | 290.x, 331.0x | N06DA, N06DX01 |
| 15 | Psychosis | 295.x, 296.1x-298.x | N05AD, N05AA, N05AB, N05AC, N05AX, N05AE, N05AF, N05AG N05AH, N05AL |
| 16 | Depression | 300.4, 301.12, 309.0x, 309.1x, 311.x | N06A |
| 17 | Bipolar disorders | 296.0x | N05AN01 |
| 18 | Anxiety | 300.0x | N05BA, N05BB01, N05CD, N05BC01, N05BC51, N05BX, N05CF, N05CX01, N06BX |
| 19 | Alcohol abuse | 291.1, 291.2, 291.5, 291.8x, 291.9, 303.9, 305.0x,  V11.3x | N07BB01 |
| 20 | Drug addiction | 292.0x, 292.82-292.89,  292.9x, 304.x, 305.2x305.9x | N07BB04 |
|  | **Diseases of the nervous system and sense organs** |  |  |
| 21 | Parkinson’s disease | 332.x | N04 |
| 22 | Multiple sclerosis | 340 | L03AB07, L03AB08, L04AA23, L04AA27, L03AX13, L04AA31, L04AA34, L03AB13, L04AX07 |
| 23 | Epilepsy | 345.x | N03AA, N03AB02, N03AB05, N03AB52, N03AX, N03AB01, N03AB04, N03AB54, N03AC01, N03AC02, N03AC03, N03AD01, N03AD02, N03AD03, N03AD51, N03AE01, N03AF01, N03AF02, N03AG01, N03AG02, N03AG03, N03AG04, N03AG05, N03AG06, N03AF03, N03AF04 |
| 24 | Glaucoma | 365.x | S01E |
|  | **Diseases of the circulatory system** |  |  |
| 25 | Ischaemic Heart Disease/Angina | 410.x – 414 | C01DA, C01DX |
| 26 | Heart failure | 428.x, 402.11, 402.91 | C01AA, C01BA93, C01BA02, C01BA01, C01BA51, C01BA71, C01DA, C03CA01 |
| 27 | Arrhythmia | 426.x, 427.x, 785.0x, | C01BC, C01BD, C01BA, C07AA07 |
| 28 | Valvular diseases | 093.20-093.24, 394.0x-397.1x, 424.00-424.91, 746.3x-746.6x | - |
| 29 | Vascular diseases | 440.x, 441.2, 441.4, 441.7, 441.9, 443.1x-443.9x, 447.1, 557.1x, 557.9x, 785.4x | - |
| 30 | Cerebrovascular diseases | 430.x-438.x | - |
| 31 | Hypertension | 401.x-405.x | C03AA, C03AB, C03AH, C03AX01, C02CA04, C03BA02, C03BA03, C03BA04, C03BA05, C03BA07, C03BA08, C03BA09, C03BA10, C03BA11, C03DB01, C03DB02, C03EA, C09BA02, C09BA03, C09BA04, C09BA05, C09BA06, C09BA07, C09BA08, C09BA09, C09BB, C09DB, C09DA01, C09DA02, C09DA03, C09DA04, C09DA06, C09DA07, C09DA08, C02AB01, C02AB02, C02AC01, C02AC02, C02AC04, C02AC05, C02DB02, C02DB03, C02DB04, C02DC01, C02DD01, C02DG01, C02KA01, C02KB01, C02KC01, C02KD01, C02KX01, C09XA |
|  | **Diseases of the respiratory system** |  |  |
| 32 | Chronic Obstructive Pulmonary Disease | 490-492.x, 494.x, 496 | R03AA, R03AB, R03AC, R03DA, R03DB, R03DA20, R01AC01, R03BC01, R01AC51, S01GX01, S01GX51, R03BA |
| 33 | Asthma | 493.x |  |
| 34 | Cystic Fibrosis | 277.0 | R05CB, R05FB01, R05FA01, A09AA02, R07AX02, R07AX30, R07AX31 |
|  | **Diseases of the digestive system** |  |  |
| 35 | Liver cirrhosis and other liver chronic diseases | 571.x, 573.x | J05AP08, J05AP09, J05AP51, J05AP53, J05AP54, J05AP55, J05AP56, J05AP57, B05AA01 |
| 36 | Inflammatory bowel diseases | 555.x-556.x | A07EC01, A07EC02, A07EC03, A07EC04 |
| 37 | Chronic and acute pancreatitis | 577.0-577.1 | - |
|  | **Diseases of the genitourinary system** |  |  |
| 37 | Kidney disease without dialysis | 582.x, 583.0, 583.1, 583.4, 583.7, 583.8, 584.6, 585.x, 586.x, 588.x | V03AE |
| 39 | Kidney dialysis with dialysis | V45.1, V56.x | - |
|  | **Diseases of the skin and subcutaneous tissues** |  |  |
| 40 | No rheumatoid psoriasis | 696.1 | D05BB01, D05BB02, D05AX |
|  | **Diseases of the musculoskeletal system and connective tissue** |  |  |
| 41 | Rheumatologic conditions (rheumatic fever, rheumatoid arthritis, Felty's syndrome, juvenile chronic polyarthritis, inflammatory spondylopathies, polymyalgia rheumatica) | 390.x, 391.x, 699.0, 714.0x, 714.1, 714.3, 714.9x, 720.0x-720.9x, 725.x | M01BA, M01CB,  P01BA02 |
| 42 | Anchylosing spondylitis | 720.0 | - |
| 43 | Systemic sclerosis | 710.1x | - |
| 44 | Systemic lupus erythematosus | 710.0x | - |
|  | **Other conditions** |  |  |
| 45 | Transplantation | V42 | L04AA01, L04AA02, L04AA03, L04AA04, L04AA05, L04AA06, L04AA08, L04AA09, L04AA10, L04AA11, L04AA12, L04AA14, L04AA15, L04AA16, L04AA17, L04AA18, L04AA19, L04AA21, L04AD01, L04AD02, L04AX01 |
| 46 | Chronic pain | 338.2, 338.4 | N02A |
| 47 | Inflammation, not elsewhere specified | - | M01A |
