# Supplementary material for: Development and validation of a clinical risk score to predict the risk of SARS-CoV-2 infection from administrative data: A population-based cohort study from Italy
Source: PLoS One. 2021 Jan 20;16(1):e0237202. doi: 10.1371/journal.pone.0237202 (PMC7816996; doi:10.1371/journal.pone.0237202)
Supplement: S3 Table — (DOCX) [file pone.0237202.s003.docx]

**S3 Table. Odds ratio (OR), and 90% confidence intervals (CI), for the relationship between selected diseases/conditions and the risk of SARS-CoV-2 infection, stratified according to gender.**

|  | **Women** | | **Men** | |
| --- | --- | --- | --- | --- |
|  | # cases /  # controls  (1,552 / 7,718) | OR (90% CI)  Independent estimates | # cases /  # controls  (1,945/ 9,640) | OR (90% CI)  Independent estimates |
| **Infectious and parasitic diseases** |  |  |  |  |
| HIV infection | 37 / 144 | 1.24 (0.91 to 1.71) | 31 / 157 | 0.89 (0.64 to 1.24) |
| **Neoplasms** |  |  |  |  |
| Malignant Neoplasms | 77 / 274 | 1.21 (0.96 to 1.52) | 78 / 387 | 0.85 (0.68 to 1.06) |
| **Endocrine, nutritional and metabolic diseases, and immunity disorders** |  |  |  |  |
| Thyroid disorders | 159 / 684 | 1.04 (0.89 to 1.23) | 66 / 236 | 1.27 (0.99 to 1.61) |
| Diabetes | 162 / 684 | 1.08 (0.91 to 1.28) | 249 / 1,048 | 1.20 (1.04 to 1.37) |
| Hyperlipidaemia | 283 / 1,495 | 0.86 (0.75 to 0.91) | 446 / 2,213 | 0.87 (0.77 to 0.97) |
| Obesity | 26 / 73 | 1.39 (0.94 to 2.07) | 22 / 80 | 1.03 (0.68 to 1.56) |
| Disorders of fluid, electrolyte, and acid-base balance | 4 / 9 | 1.37 (0.47 to 4.05) | 4 / 20 | 0.74 (0.30 to 1.87) |
| Hyperuricemia/Gout | 68 / 265 | 0.97 (0.74 to 1.25) | 112 / 446 | 1.17 (0.96 to 1.42) |
| **Diseases of the blood and blood-forming organs** |  |  |  |  |
| Coagulation defects | 3 / 7 | 1.44 (0.45 to 4.61) | 3 / 17 | 0.72 (0.25 to 2.04) |
| Anaemias | 161 / 521 | 1.33 (1.12 to 1.58) | 104 / 406 | 1.10 (0.90 to 1.36) |
| **Mental disorders** |  |  |  |  |
| Dementia / Alzheimer | 29 / 54 | 2.03 (1.32 to 3.12) | 19 / 35 | 2.46 (1.47 to 4.12) |
| Psychosis | 79 / 158 | 1.91 (1.46 to 2.50) | 45 / 145 | 1.43 (1.05 to 1.95) |
| Depression | 150 / 599 | 1.03 (0.84 to 1.26) | 83 / 404 | 0.91 (0.73 to 1.13) |
| Bipolar disorders | 7 / 12 | 1.90 (0.85 to 4.25) | 1 / 17 | 0.27 (0.05 to 1.48) |
| Anxiety | 646 / 2,674 | 1.17 (1.06 to 1.31) | 723 / 2,941 | 1.33 (1.20 to 1.47) |
| **Diseases of the nervous system and sense organs** |  |  |  |  |
| Parkinson’s disease | 31 / 84 | 1.27 (0.87 to 1.86) | 36 / 104 | 1.44 (1.02 to 2.04) |
| Multiple sclerosis | 4 / 11 | 1.56 (0.57 to 4.28) | 1 / 13 | 0.37 (0.07 to 2.05) |
| Epilepsy | 106 / 329 | 1.28 (1.04 to 1.58) | 70 / 331 | 0.94 (0.74 to 1.19) |
| Glaucoma | 52 / 226 | 1.19 (0.91 to 1.56) | 67 / 256 | 1.25 (0.99 to 1.59) |
| **Diseases of the circulatory system** |  |  |  |  |
| Ischaemic Heart Disease/Angina | 60 / 250 | 0.78 (0.58 to 1.03) | 153 / 591 | 1.10 (0.91 to 1.34) |
| Heart failure | 157 / 479 | 1.72 (1.39 to 2.12) | 124 / 526 | 0.92 (0.75 to 1.13) |
| Arrhythmia | 72 / 301 | 0.95 (0.73 to 1.22) | 124 / 437 | 1.31 (1.08 to 1.59) |
| Valvular diseases | 18 / 78 | 0.72 (0.44 to 1.18) | 25 / 102 | 0.87 (0.59 to 1.29) |
| Vascular diseases | 14 / 45 | 1.06 (0.62 to 1.83) | 38 / 141 | 1.00 (0.72 to 1.39) |
| Cerebrovascular diseases | 57 / 170 | 1.11 (0.82 to 1.49) | 70 /275 | 0.95 (0.74 to 1.23) |
| Hypertension | 356 / 1,600 | 1.10 (0.97 to 1.25) | 470 / 2,131 | 1.05 (0.94 to 1.18) |
| **Diseases of the respiratory system** |  |  |  |  |
| Chronic Pulmonary Disease | 106 / 383 | 1.18 (0.97 to 1.44) | 137 / 525 | 1.19 (1.00 to 1.42) |
| Cystic Fibrosis | 5 / 9 | 3.35 (1.24 to 9.09) | 3 / 25 | 0.58 (0.21 to 1.62) |
| **Diseases of the digestive system** |  |  |  |  |
| Liver cirrhosis and other liver chronic diseases | 22 / 77 | 0.94 (0.61 to 1.44) | 32 / 139 | 0.92 (0.65 to 1.29) |
| Inflammatory bowel diseases | 23 / 69 | 1.49 (0.98 to 2.25) | 31 / 100 | 1.45 (1.03 to 2.04) |
| Chronic and acute pancreatitis | 5 / 27 | 0.64 (0.26 to 1.56) | 5 / 29 | 0.74 (0.33 to 1.71) |
| **Diseases of the genitourinary system** |  |  |  |  |
| Kidney disease with or without dialysis | 29 / 62 | 1.59 (1.06 to 2.41) | 38 / 148 | 0.91 (0.64 to 1.28) |
| **Diseases of the skin and subcutaneous tissues** |  |  |  |  |
| Psoriasis | 10 / 41 | 1.03 (0.57 to 1.87) | 13 / 72 | 0.87 (0.52 to 1.45) |
| **Diseases of the musculoskeletal system and connective tissue** |  |  |  |  |
| Rheumatologic conditions | 16 / 55 | 1.26 (0.77 to 2.04) | 12 / 24 | 2.11 (1.16 to 3.83) |
| **Other conditions** |  |  |  |  |
| Transplantation | 6 / 23 | 0.98 (0.44 to 2.14) | 7 / 36 | 0.86 (0.42 to 1.73) |
| Chronic pain | 50 / 229 | 0.92 (0.70 to 1.22) | 39 / 149 | 1.30 (0.95 to 1.78) |
| Inflammation, not elsewhere specified | 221 / 1,195 | 0.85 (0.74 to 0.98) | 189 / 1,049 | 0.84 (0.73 o 0.97) |
