# Supplementary material for: Development and validation of a clinical risk score to predict the risk of SARS-CoV-2 infection from administrative data: A population-based cohort study from Italy
Source: PLoS One. 2021 Jan 20;16(1):e0237202. doi: 10.1371/journal.pone.0237202 (PMC7816996; doi:10.1371/journal.pone.0237202)
Supplement: S4 Table — (DOCX) [file pone.0237202.s004.docx]

**S4 Table.** **Odds ratio (OR), and 90% confidence intervals (CI), for the relationship between selected diseases/conditions and the risk of SARS-CoV-2 infection, stratified according to age categories (i.e., younger and older 65 years)**.

|  | **< 65 years** | | **≥ 65 years** | |
| --- | --- | --- | --- | --- |
|  | # cases /  # controls  (2,375 / 11,820) | OR (90% CI)  Independent estimates | # cases /  # controls  (1,122 / 5,538) | OR (90% CI)  Independent estimates |
| **Infectious and parasitic diseases** |  |  |  |  |
| HIV infection | 36 / 185 | 0.94 (0.69 to 1.27) | 32 / 116 | 1.25 (0.89 to 1.76) |
| **Neoplasms** |  |  |  |  |
| Neoplasms | 66 / 243 | 1.15 (0.90 to 1.47) | 89 / 418 | 0.96 (0.77 to 1.19) |
| **Endocrine, nutritional and metabolic diseases, and immunity disorders** |  |  |  |  |
| Thyroid disorders | 115 / 522 | 1.05 (0.88 to 1.26) | 110 / 398 | 1.24 (1.01 to 1.51) |
| Diabetes | 136 / 585 | 1.19 (1.00 to 1.43) | 275 / 1,147 | 1.13 (0.99 to 1.30) |
| Hyperlipidaemia | 274 / 1,429 | 0.89 (0.77 to 1.01) | 455 / 2,279 | 0.88 (0.77 to 0.99) |
| Obesity | 29 / 102 | 1.17 (0.81 to 1.69) | 19 / 51 | 1.33 (0.83 to 2.13) |
| Disorders of fluid, electrolyte, and acid-base balance | 3 / 11 | 0.81 (0.25 to 2.61) | 5 / 18 | 0.82 (0.33 to 1.99) |
| Hyperuricemia/Gout | 35 / 166 | 0.93 (0.67 to 1.29) | 145 / 545 | 1.10 (0.92 to 1.32) |
| **Diseases of the blood and blood-forming organs** |  |  |  |  |
| Coagulation defects | 3 / 12 | 0.92 (0.31 to 2.72) | 3 / 12 | 0.80 (0.27 to 2.37) |
| Anaemias | 99 / 375 | 1.22 (0.99 to 1.49) | 166 / 552 | 1.28 (1.07 to 1.53) |
| **Mental disorders** |  |  |  |  |
| Dementia / Alzheimer | 4 / 4 | 5.35 (1.61 to 17.76) | 44 / 85 | 1.78 (1.26 to 2.51) |
| Psychosis | 24 / 158 | 0.47 (0.28 to 0.79) | 100 / 145 | 2.94 (2.28 to 3.78) |
| Depression | 86 / 479 | 0.84 (0.68 to 1.04) | 147 / 524 | 1.12 (0.94 to 1.34) |
| Bipolar disorders | 6 / 21 | 1.75 (0.79 to 3.86) | 2 / 8 | 0.60 (0.16 to 2.30) |
| Anxiety | 790 / 3,205 | 1.32 (1.21 to 1.44) | 579 / 2,410 | 1.16 (1.01 to 1.32) |
| **Diseases of the nervous system and sense organs** |  |  |  |  |
| Parkinson’s disease | 12 / 33 | 2.41 (1.31 to 4.41) | 55 / 155 | 1.18 (0.89 to 1.58) |
| Multiple sclerosis | 5 / 24 | 0.93 (0.40 to 2.14) | 0 / 0 | - |
| Epilepsy | 70 / 346 | 0.98 (0.77 to 1.24) | 314 / 106 | 1.33 (1.08 to 1.64) |
| Glaucoma | 37 / 143 | 1.22 (0.89 to 1.68) | 82 / 339 | 1.23 (0.99 to 1.53) |
| **Diseases of the circulatory system** |  |  |  |  |
| Ischaemic Heart Disease/Angina | 52 / 237 | 0.98 (0.73 to 1.31) | 161 / 604 | 1.02 (0.84 to 1.24) |
| Heart failure | 26 / 160 | 0.62 (0.42 to 0.91) | 255 / 845 | 1.36 (1.15 to 1.60) |
| Arrhythmia | 54 / 158 | 1.65 (1.25 to 2.17) | 142 / 580 | 1.01 (0.84 to 1.21) |
| Valvular diseases | 11 / 58 | 0.78 (0.44 to 1.39) | 32 / 122 | 0.87 (0.60 to 1.27) |
| Vascular diseases | 12 / 55 | 0.78 (0.44 to 1.37) | 40 / 131 | 1.09 (0.78 to 1.51) |
| Cerebrovascular diseases | 17 / 102 | 0.67 (0.42 to 1.05) | 110 / 343 | 1.08 (0.87 to 1.35) |
| Hypertension | 323 / 1,435 | 1.11 (0.98 to 1.25) | 503 / 2,296 | 1.06 (0.94 to 1.19) |
| **Diseases of the respiratory system** |  |  |  |  |
| Chronic Pulmonary Disease | 91 / 363 | 1.23 (1.00 to 1.50) | 153 / 545 | 1.17 (0.98 to 1.39) |
| Cystic Fibrosis | 3 / 16 | 0.94 (0.31 to 2.83) | 5 / 18 | 1.45 (0.60 to 3.49) |
| **Diseases of the digestive system** |  |  |  |  |
| Liver cirrhosis and other liver chronic diseases | 24 / 114 | 0.85 (0.57 to 1.26) | 30 / 102 | 1.12 (0.78 to 1.62) |
| Inflammatory bowel diseases | 38 / 98 | 1.86 (1.35 to 2.56) | 16 / 71 | 1.05 (0.66 to 1.68) |
| Chronic and acute pancreatitis | 3 / 21 | 0.61 (0.21 to 1.79) | 7 / 35 | 0.87 (0.43 to 1.77) |
| **Diseases of the genitourinary system** |  |  |  |  |
| Kidney disease with or without dialysis | 17 / 49 | 1.48 (0.88 to 2.47) | 50 / 161 | 0.99 (0.73 to 1.35) |
| **Diseases of the skin and subcutaneous tissues** |  |  |  |  |
| Psoriasis | 11 / 63 | 0.85 (0.50 to 1.47) | 12 / 50 | 1.00 (0.57 to 1.73) |
| **Diseases of the musculoskeletal system and connective tissue** |  |  |  |  |
| Rheumatologic conditions | 12 / 39 | 1.53 (0.88 to 2.68) | 16 / 40 | 1.65 (0.99 to 2.74) |
| **Other conditions** |  |  |  |  |
| Transplantation | 7 / 34 | 0.75 (0.36 to 1.56) | 6 / 25 | 1.00 (0.46 to 2.17) |
| Chronic pain | 28 / 116 | 1.22 (0.85 to 1.76) | 61 / 262 | 0.99 (0.77 to 1.28) |
| Inflammation, not elsewhere specified | 174 / 1,140 | 0.70 (0.60 to 0.81) | 236 / 1,104 | 1.04 (0.90 to 1.19) |
